# Effect of Exosomal lncRNA MALAT1/miR-370-3p/STAT3 Positive Feedback Loop on PI3K/Akt Pathway Mediating Cisplatin Resistance in Cervical Cancer Cells

**DOI:** 10.1155/2023/6341011

**Published:** 2023-02-06

**Authors:** Yi Hu, Genlin Li, Yan Ma, Guifang Luo, Qian Wang, Shufen Zhang

**Affiliations:** Department of Obstetrics and Gynaecology, The First Affiliated Hospital of Hengyang Medical School, University of South China, Hengyang, Hunan 421001, China

## Abstract

**Background:**

Exosomes can encapsulate lncRNA to mediate intercellular communication in cancer progression. Our study devoted to research the effect that long noncoding RNA Metastasis-associated lung adenocarcinoma transcript 1 (lncRNA MALAT1) influence on cervical cancer (CC).

**Methods:**

MALAT1 and miR-370-3p levels in CC was assessed using qRT-PCR. CCK-8 assay and flow cytometry were devoted to confirm the influence on MALAT1 influencing the proliferation in cisplatin-resistant CC cells. Futher more, MALAT1, combined with miR-370-3p was confirmed by dual-luciferase reporter assay and RNA immunoprecipitation assay.

**Results:**

In CC tissues, MALAT1 turned into substantially expressed, cisplatin-resistant cell lines, as well as exosomes. Cell proliferation was restrained and cisplatin-induced apoptosis was promoted by way of Knockout MALAT1. And promoted the miR-370-3p level, MALAT1 targeted miR-370-3p. Promoting effect of MALAT1 on cisplatin resistance of CC was partially reversed through miR-370-3p. In addition, STAT3 may induce up-regulation of MALAT1 expression in cisplatin-resistant CC cells. It was further confirmed that the effect of MALAT1 on cisplatin-resistant CC cells was achieved by activating PI3K/Akt pathway.

**Conclusion:**

The positive feedback loop of exosomal MALAT1/miR-370-3p/STAT3 mediates the cisplatin resistance of cervical cancer cells affecting PI3K/Akt pathway. Exosomal MALAT1 may become a promising therapeutic target for treating cervical cancer.

## 1. Introduction

Cervical cancer (CC) is the fourth leading cause of cancer death in women [1]. Despite the rapid development of prevention and treatment strategies in the past few decades, the prognosis of patients with advanced or recurrent cervical cancer is still poor [2]. CC has a high sensitivity to chemotherapy. Chemotherapy is one of the most commonly used methods for treating advanced or recurrent cervical cancer. A study has shown that cisplatin is one of the most effective drugs for cervical cancer [3]. It has been confirmed that various signal transduction pathways play a vital part in the molecular mechanism of its anticancer effect [4, 5]. At present, the biggest challenge to improve the long-term efficacy of cervical cancer is cisplatin resistance, and its mechanism is not precise.

Polyvesicles in the cellular endocytosis pathway form vesicles through invagination of the cell membrane to form vesicles and release them into the extracellular matrix. Polyvesicles mediate proteins, nucleic acids, and lipids wrapped by exocrine bodies to participate in the process of intercellular information transmission [6]. Long noncoding RNA (lncRNA) regulates gene expression in receptor cells mediated by exocrine and then exert their biological effects in a variety of ways [7]. Whether exosomes come from drug-resistant cancer cells can make sensitive cells resistant remains to be clarified.

lncRNA may be a potential driving force for the development of cancer and can assess the bad prognosis of cancer, a large amount of studies have proved [8, 9]. Studies have revealed that lncRNA Metastasis-associated lung adenocarcinoma transcript 1 (MALAT1) affects the biological activity of various tumors by interacting with DNA, mRNA, miRNA, and proteins. Some studies have confirmed that OCT4, as a transcription factor, can induce up-regulate MALAT1 expression. MALAT1 can promote cell proliferation of lung cancer [10]. LncRNAs are of vital importance in drug resistance of tumors, which has been confirmed. DILA1 is a specific therapeutic target for down-regulating cyclin D1 and reversing tamoxifen resistance in breast cancer [11]. In addition, MALAT1 can activate PI3K/AKT pathway and promote cisplatin resistance in cervical cancer [12]. Moreover, previous study has confirmed that MALAT1 transfection mediated by exosomes can induce the proliferation of breast cancer cells [13]. However, the role of exosomal MALAT1 in cisplatin resistance of CC has not been studied.

The expression of microRNA (miRNA) is different between normal cells and malignant cells, and there is an abnormal expression of miRNA in almost all tumors [14, 15]. Previous studies have confirmed that miR-370-3p/RAF1 axis could inhibit the cervical cancer survival [16]. Moreover, in LPS-induced acute renal injury, MALAT1 targeting miR-370-3p attenuates injury [17]. However, in cisplatin resistance in cervical cancer, the role of MALAT1/miR-370-3p role is not expounded. Furthermore, STAT3 accelerates drug resistance of cervical cancer cells [18]. STAT3 is raised and the resistance of ovarian cancer cells to cisplatin is reduced by PI3K/AKT pathway [19]. Given the above research basis, present research assessed the influence that exosomal MALAT1 on cisplatin resistance in CC cells. Furthermore, we also made a thorough inquiry into the MALAT1/miR-370-3p/STAT3 regulatory network and discussed the therapeutic significance of external MALAT1 in patients with CC.

## 2. Materials and Methods

### 2.1. Clinical Samples of CC Patients

From 2017 June to 2020 May, 80 pairs of CC and paracancerous typical tissues had been acquired within the hospital. None of the patients recruited for this study received preoperative radiotherapy or chemotherapy. Otherwise, it was assessed as invasive carcinoma using pathologists. To better continuously reassess patients, the histological kind and grade of most cancers cell differentiation had been re-evaluated and determined in line with the revised World Health Organization category gadget in 2004 [20], and the postoperative pathological degree was determined consistent with the international staging device. Corresponding adjacent tissues and tumor samples were gathered, stored and used. To purify extracellular bodies, serum samples were gathered from those 80 patients.

### 2.2. Cell Culture

Human cervical epithelial cells (HcerEpic) and CC cell lines (Hela and C33A) were bought from CellBank (Shanghai, China). Induction of cervical cancer cisplatin cell line: cisplatin-resistant CC cell line was produced by low concentration dose continuous induction. Hela and C33A cells were dealt with 0.1 *μ*g/ml cisplatin for 2 weeks, 0.5 *μ*g/ml for 6 weeks, 1 *μ*g/ml for 12weeks, 2 *μ*g/ml for 8 weeks, and 4 *μ*g/ml for 12weeks. After 40 weeks of induction, cervical cancer cells could grow stably and passage generally in the culture medium containing cisplatin 4 *μ*g/ml. Hela/R and C33A/R cell lines were successfully established. In a hatchery at 5% CO_2_, 37°C, in DMEM medium, the cells were cultured and purified, medium contains 5% FBS. The liquid was changed every 2 days and passaged once in 5 days, and the experiment was performed while the cells grew to around 90%.

### 2.3. Cell Transfection

The cells in the logarithmic growth phase were digested with trypsin and counted. 5 × 10^4^ cells were inoculated in each well and incubated in the incubator to 70% abundance, pcDNA 3.1-MALAT1/si-MALAT1 and lncRNA-NC (negative control), miR-370-3p mimics/inhibitors, and miR-NC (negative control) 100 pmol were mixed with liposomes and placed in 30 min at room temperature.

At the same time, the cells were rinsed with serum-free medium 2 times, and the mixed liposomes were diluted with serum-free medium and carefully added to the rinsed cells. Then the transfected cells were incubated in an incubator for 24 h and replaced with a completely fresh medium. The specific operation was carried out according to the Lipofectamine2000 (Thermo Fisher) manual.

### 2.4. Extraction of Exosome

The cells were inoculated in a Petri dish and cultured in the exocrine-free medium for 2 days, and then the supernatant was collected. Supernatant 300 × *g* centrifugation for 10 min, removed the cells, and then 2000 × *g* centrifugation for 20 min, removed the cell fragments. Later, the supernatant was centrifuged for 1.1 h by 10000 × *g*, removed the precipitation. After 100000 × *g* ultracentrifugation 70 min, the precipitates were collected and re-suspended with 50∼100 *μ*L PBS, stored at 4°C or −80°C.

### 2.5. QRT-PCR

The transfected were collected, all RNA was gotten from the samples by TRIzol method, RNA concentration was determined spectrophotometrically and cDNA was synthesized by reverse transcription. QRT-PCR was performed according to SYBRPremix ExTaqTM II kit instructions (TaKaTa, Japan). Reaction conditions: 95°C, 0.5 min; 95°C, 5 s, 58°C, 0.5 min, 40 cycles; 95°C, 15 s, 58°C, 0.5 min, 95°C, 15 s. Using U6 and GAPDH as internal reference, the 2^−ΔΔCt^ way was utilized to get miR-370-3p expression levels and LncRNA MALAT1, STAT3, miR-370-3p levels. All primer sequences were synthesized by Guangzhou Ribo Bio (China). See Table 1.

### 2.6. CCK-8 Assay

In the logarithmic development stage, Hela/R and C33A/R cells were digested. In the wake of changing the cell density to 2 × 10^3^/mL, they were inoculated 100 *μ*L per hole cell suspension was cultured in 96 well plate with 3 wells in each group. After 24 h, twelve*μ*L CCK-8 reagent (Yisheng Biotechnology) was supplied to each hole, cultured for 60 min. After the end of the culture, within the enzyme labeling instrument, the 96-well plate was placed, and the absorbance of each well was measured at 450 nm wavelength.

### 2.7. Flow Cytometry

Collected the transfected cells 1 × 10^6^, suspended at 500 *μ*L in the binding buffer, and then added 5 *μ*L annexin V—fluorescein isothiocyanate (FITC) and 5 *μ*L propidium iodide (PI) was placed in the dark for 20 min at room temperature, and the apoptosis was analyzed by flow cytometry.

### 2.8. Dual-Luciferase Reporter Assay

StarBase 3.0 predicted target binding sites between MALAT1 and miR-370-3p, and TargetScan 7. 1 predicted target common site between miR-370-3p and STAT3. Wild-type (WT) and mutant (MUT) reporter gene plasmids were performed against the MALAT1 3′-UTR end and STAT3 3′-UTR end sequences. MALAT1-WT and MALAT1-MUT (or STAT3-WT and STAT3-MUT) were co-transfected into HEK-293T cells with NC-mimics and miR-370-3p mimics, separately, and luciferase activity was assayed after 48 h.

### 2.9. RNA Immunoprecipitation (RIP) Assay

Use Magna rip Kit (millipore) and follow the instructions. Cells were collected and lysed with RIP lysis buffer; Then, Anti coated anti human Ago2 antibody magnetic beads were used for ago2 immunoprecipitation, and IgG antibody was used as negative control. Then, immunoprecipitated RNA was isolated, and the abundance of MALAT1 and miR-370-3p in the binding part was detected by QRT-PCR.

### 2.10. Western Blot

The transfected cells were gotten and the protein level was identified by BCA approach. SDS-PAGE gel electrophoresis seperated 30 *μ*g of protein, which was transferred to PVDF membrane. 10% skimmed milk powder was closed at the temperature of room for 3 hours. Primary antibody were put and cultivated under 4°C all the night. HRP-labelled secondary antibodies (1 : 2500) were cultivated at the temperature of room for 1 h. The band was analysed by Image J, and gray level of the targeted protein was expressed as the proportation of the gray value of the targeted protein band to the gray value of the GAPDH band. All antibodies were bought from Abcam (UK).

### 2.11. Statistical Analysis

This study used the SPSS 20.0 statistical software to analyze. Statistical data were shown as average ± SD, and one-way ANOVA was utilized to make a comparison between groups, and LSD experiment was utilized for two-way comparison; test level *α* = 0.05.

## 3. Results

### 3.1. MALAT1 was Raised in Cisplatin-Resistant CC Cells

**Figure 1:**
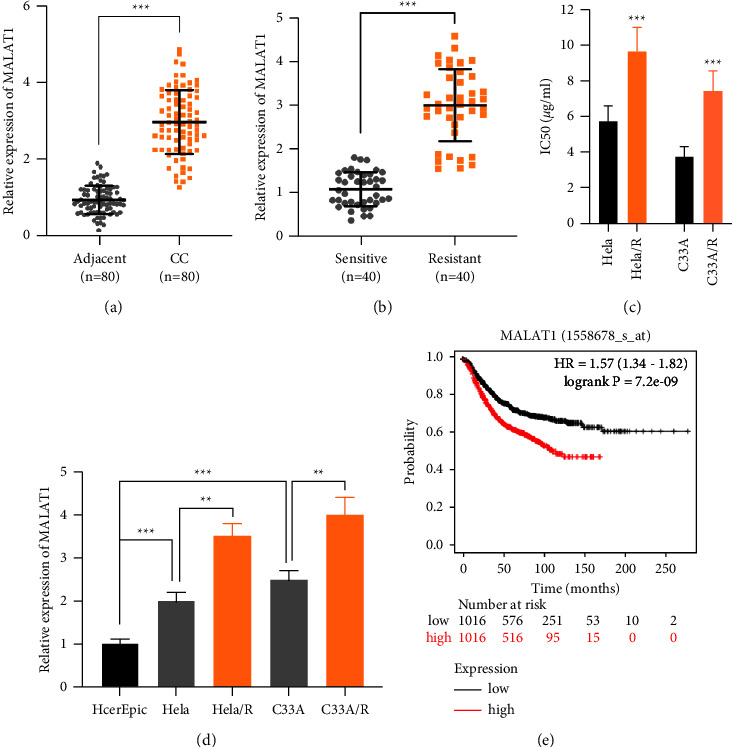
MALAT1 was upregulated in cisplatin-resistant CC cells. (a) The expression of MALAT1 in CC patients and paracancerous cervical tissues. (b) The relative expression of MALAT1 in cisplatin-sensitive group and cisplatin-resistant group in CC patients. (c) Cell viability was assessed at varying concentrations of cisplatin to calculate IC_50_ values. (d) The relative expression of MALAT1 in CC cell lines. (e) Kaplan-Meier analyzed the correlation between MALAT1 expression and overall survival rate in patients with CC from TCGA CC data sets. All the tests were carried out at least three times. ^*∗∗*^*P* < 0.01, ^*∗∗∗*^*P* < 0.001. All experiments were performed at least three times.

Compared with adjacent cervical tissues, MALAT1 was highly expressed in CC tissues (Figure 1(a)). What is more, in cisplatin-resistant CC patients, MALAT1 was raised (*n* = 40) compared with CC patients who were sensitive to Cisplatin chemotherapy (*n* = 40) (Figure 1(b)). Using repeated cisplatin incubation to establish cisplatin-resistant Hela and C33A cells. Cisplatin-resistant Hela and C33A cells had much higher IC_50_ values for cisplatin than the parental Hela and C33A cells (Figure 1(c)). Further detection of the level of MALAT1 in CC cells was higher than that in HcerEpic cells. MALAT1 was significantly increased in cisplatin-resistant Hela/R and C33A/R cells in these CC cells (Figure 1(d)). Tumor size, tumor stage, and distant metastasis was correlated with MALAT1 high level (Table 2). Moreover, Kaplan-Meier survival analysis from TCGA CC data sets indicated MALAT1 high level was memorably correlated with bad overall survival (OS) in CC tissues (Figure 1(e)). Therefore, MALAT1 might does a impact part in the drug resistance of CC cells to MALAT1, and high levels of MALAT1 might predict the poor prognosis of CC.

### 3.2. Effects of MALAT1 on Proliferation and Apoptosis of Drug-Resistant CC Cell Line

**Figure 2:**
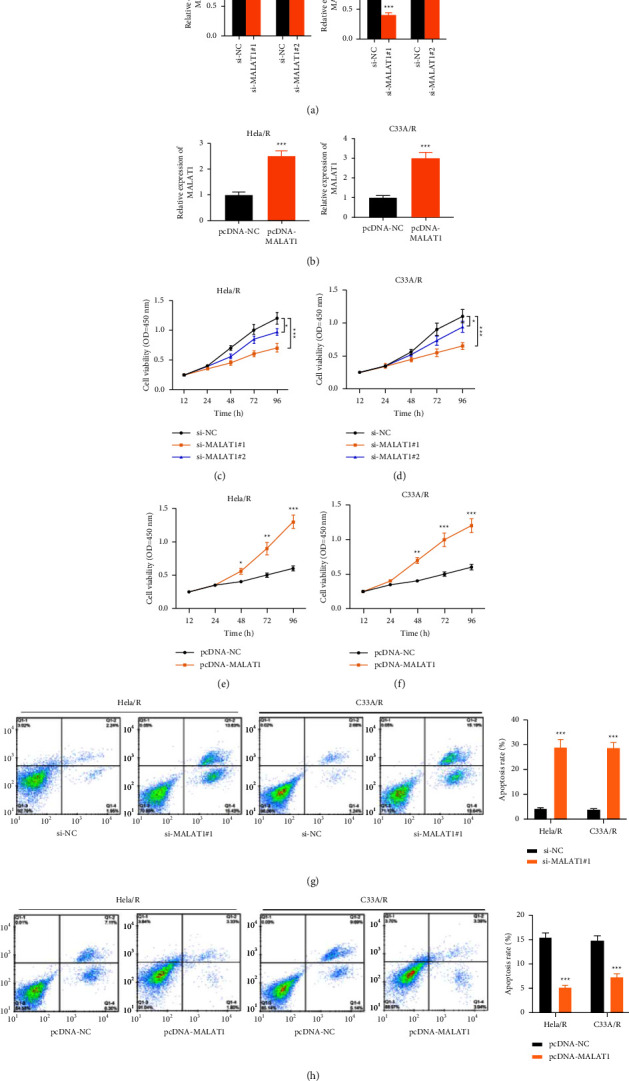
Effect of MALAT1 on proliferation and apoptosis of drug-resistant CC cell lines. We used the si-MALAT1 expression vector and pcDNA-MALAT1 expression vector to transfect Hela/R and C33A/R cell lines to induce ectopic expression of MALAT1. (a, b) The transfection efficiency was detected by qRT-PCR. (c–f) CCK-8 assay was used to detect cell proliferation. (g, h) Apoptosis was detected by flow cytometry. ^*∗*^*P* < 0.05, ^*∗∗*^*P* < 0.01, ^*∗∗∗*^*P* < 0.001. All experiments were performed at least three times.

It was found that the MALAT1 was remarkably reduced by specific siRNA (Figure 2(a)). At the same time, we used the pcDNA-MALAT1 expression vector to transfect Hela/R and C33A/R cell lines to induce ectopic overexpression of MALAT1 (Figure 2(b)). MALAT1 silencing inhibited the proliferation of cisplatin drug-resistant cells while the concentration of cisplatin was 2 *μ*g/ml, (Figures 2(c) and 2(d)). Whereas, after MALAT1 overexpression, the development rate of Hela/R and C33A/R cells was considerably increased (Figures 2(e) and 2(f)). These indicated that MALAT1 promoted the proliferation of cisplatin-resistant CC cells. Apoptosis rate was analyzed the by flow cytometry. Silencing MALAT1 promoted the cisplatin exposed CC cells apoptosis (Figure 2(g)). After overexpression of MALAT1, the apoptosis rate of Hela/R and C33A/R cells treated with 2 *μ*g/ml cisplatin was notably decreased (Figure 2(h)).

### 3.3. STAT3 Induced the Expression of MALAT1 in Cisplatin-Resistant CC Cells

**Figure 3:**
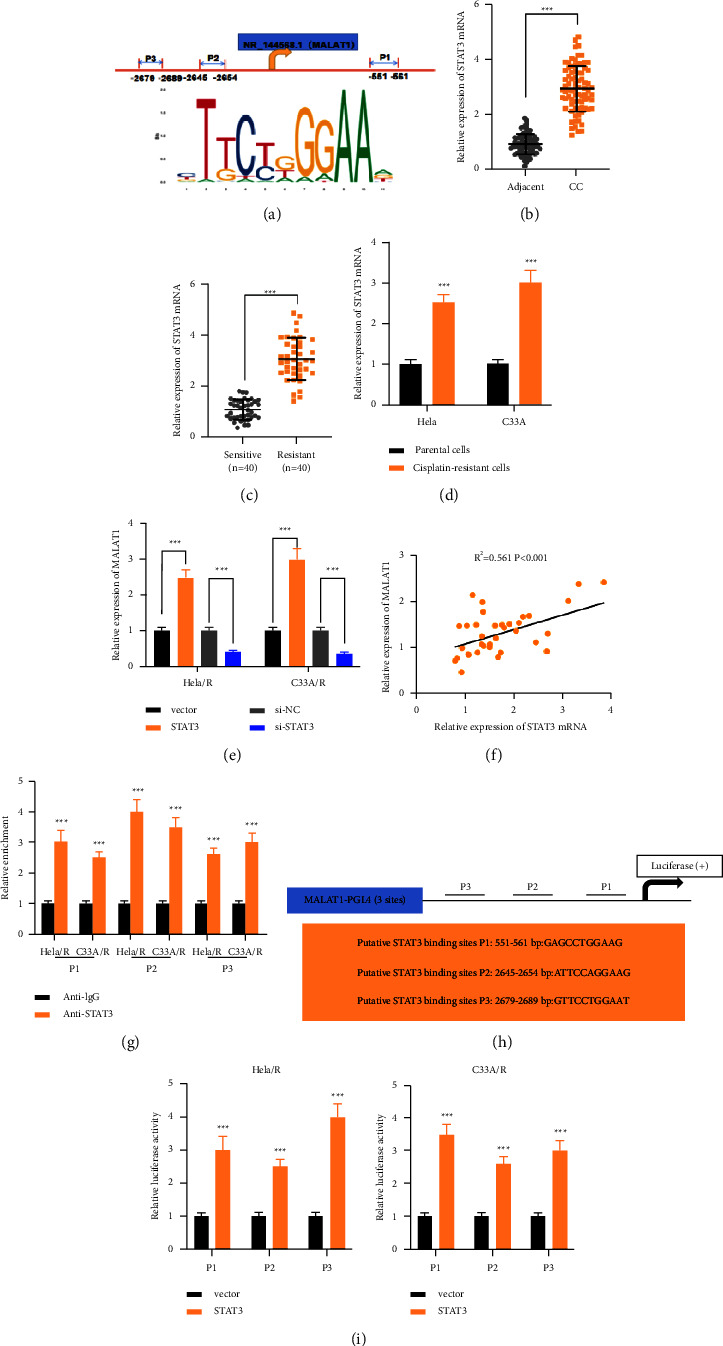
STAT3 induced the expression of MALAT1 in cisplatin-resistant CC cells. (a) Jaspar was used to predict the STAT3 binding site of the MALAT1 promoter region. (b) qRT-PCR was used to detect the level of STAT3 in patients with CC and adjacent cervical tissues. (c) The relative expression of STAT3 in cisplatin-sensitive group and cisplatin-resistant group in CC patients. (d) The expression of STAT3 in drug-resistant cells and parent cells was detected by qRT-PCR. (e) After low or high expression of STAT3, the expression of MALAT1 in CC cisplatin-resistant cells was detected by qRT-PCR. (f) Pearson's analysis was used to analyze the correlation between STAT3 and MALAT1 in CC-resistant tissues. (g) STAT3 binding sites were analyzed by ChIP assay. (h) Schematic diagram of luciferase reporter plasmid constructed. (i) The relative luciferase activity was detected by dual-luciferase reporter assay. ^*∗∗∗*^*P* < 0.001. All experiments were performed at least three times.

There is growing evidence that several key transcription factors cause lncRNA disorders in human cancer cells [21]. Therefore, we explored whether transcription factors can regulate MALAT1. JASPAR (https://jaspar.genereg.net/) was applied, several STAT3 binding sites in the MALAT1 promoter region with high scores (Figure 3(a)). STAT3 was more highly expressed in CC tissues than in normal cervical tissues (Figure 3(b)). Besides, the expression of STAT3 was up-regulated in cisplatin-resistant CC patients compared with CC patients who were sensitive to Cisplatin chemotherapy (Figure 3(c)). The expression of STAT3 in cisplatin-resistant CC cells was up-regulated compared with parent cells at the transcriptional and protein levels (Figure 3(d)). Besides, transfection of STAT3 overexpression vector significantly increased the expression of MALAT1, while in two cisplatin drug-resistant cell lines, STAT3 knockout induced a decrease in MALAT1 expression (Figure 3(e)). Pearson's analysis showed a positive correlation between MALAT1 and STAT3 at the level of mRNA in the samples of CC patients who were clinically resistant to cisplatin (Figure 3(f)). We also carried out ChIP analysis to further verify the enrichment of STAT3 in the promoter region of MALAT1 (Figure 3(g)). Furthermore, the MALAT1 promoter region containing three potential binding sites of STAT3 was inserted into the PGL4 luciferase reporter vector (Figure 3(h)). STAT3 enhanced the luciferase activity (Figure 3(i)). These results indicated that MALAT1 was upregulated in cisplatin-resistant CC cells induced by STAT3.

### 3.4. MiR-370-3p was the Downstream miRNA of MALAT1

**Figure 4:**
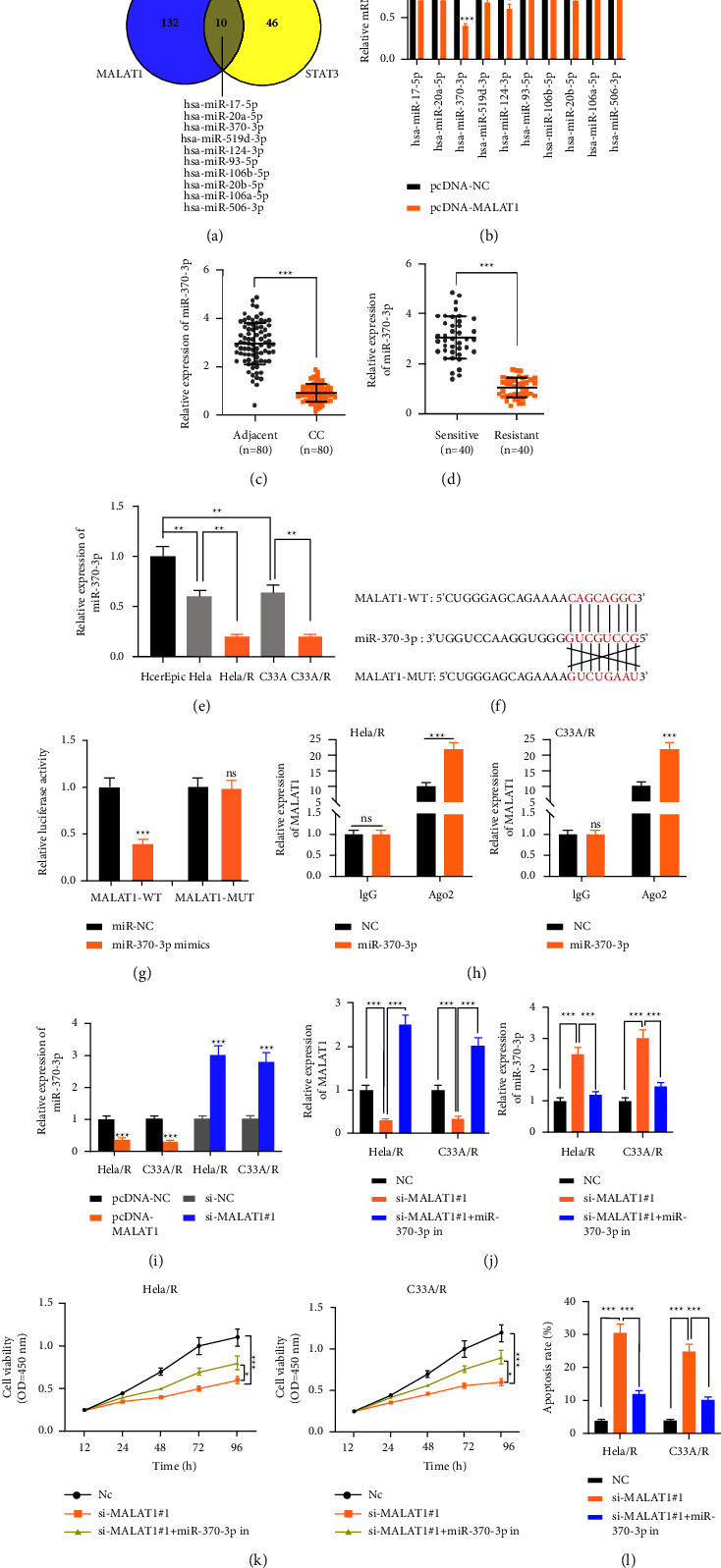
miR-370-3p was the downstream miRNA of MALAT1 and was negatively regulated by MALAT1. (a) The database showed that miRNAs had conserved binding sites with MALAT1 and STAT3. (b) The expression of miR-370-3p after overexpression of MALAT1. (c) The expression of miR-370-3p in CC patients and paracancerous cervical tissues. (d) The relative expression of MALAT1 in cisplatin-sensitive group and cisplatin-resistant group in CC patients. (e) The relative expression of miR-370-3p in CC cell lines was detected by qRT-PCR. (f) There were conservative binding sites between MALAT1 and miR-370-3p. (g) Dual-luciferase reporter assay was used to detect the binding of MALAT1 to miR-370-3p. (h) RIP assay was used to detect the binding of MALAT1 and miR-370-3p. (i) The effect of MALAT1 on miR-370-3p was detected by qRT-PCR. Hela/R and C33A/R cells were co-transfected with MALAT1 low expression plasmid and miR-370-3p inhibitors. (j) qRT-PCR was used to detect the expression of MALAT1 and miR-370-3p. (k) CCK-8 assay was used to detect cell proliferation. (l) apoptosis was detected by flow cytometry. ^*∗*^*P* < 0.05, ^*∗∗*^*P* < 0.01, ^*∗∗∗*^*P* < 0.001. All experiments were performed at least three times.

From StarBase, 10 miRNAs have conserved binding sites to MALAT1 and STAT3 (Figure 4(a)). miR-370-3p expression was significantly down-regulated (Figure 4(b)). The results of qRT-PCR showed that the expression of miR-370-3p in CC tissues was significantly lower than that in paracancerous tissues (Figure 4(c)). Moreover, the miR-370-3p expression was decreased in patients with cisplatin-resistant CC (*n* = 40) compared with those in CC patients who were sensitive to cisplatin chemotherapy (*n* = 40) (Figure 4(d)). The expression of miR-370-3p in CC cell lines was lower than that in HcerEpic cells. In these CC cells, the expression of miR-370-3p in Cisplatin-resistant Hela/R and C33A/R cells decreased dramatically (Figure 4(e)). miR-370-3p contained complementary binding sequences of MALAT1 (Figure 4(f)). The effect of miR-370-3p on WT-MALAT1 and MUT-MALAT1 luciferase reporter system was measured. Compared with the negative control, miR-370-3p markedly inhibited the activity of the WT-MALAT1 luciferase reporter vector, while miR-370-3p did not affect the luciferase activity of MUT-MALAT1 transfected HEK293 (Figure 4(g)). Both MALAT1 and miR-370-3p existed in the products of anti-AGO2 precipitation (Figure 4(h)), indicating that miR-370-3p is a MALAT1-targeted miRNA. Overexpressed or silenced MALAT1 down-regulated and up-regulated miR-370-3p expression, respectively (Figure 4(i)). Subsequently, NC (siRNA targeting MALAT1), MALAT1 low expression plasmid, and miR-370-3p inhibitor were co-transfected into cells, and the transfection efficiency was detected (Figure 4(j)). The results showed that miR-370-3p could reverse the effect of MALAT1 on promoting activity and inhibiting apoptosis in CC-resistant cells (Figures 4(k) and 4(l)). The above results revealed that miR-370-3p was the target of MALAT1, and could incompletely reverse the impact of MALAT1 on the proliferation of CC cisplatin-resistant cells.

### 3.5. STAT3 was the Target of miR-370-3p in CC

**Figure 5:**
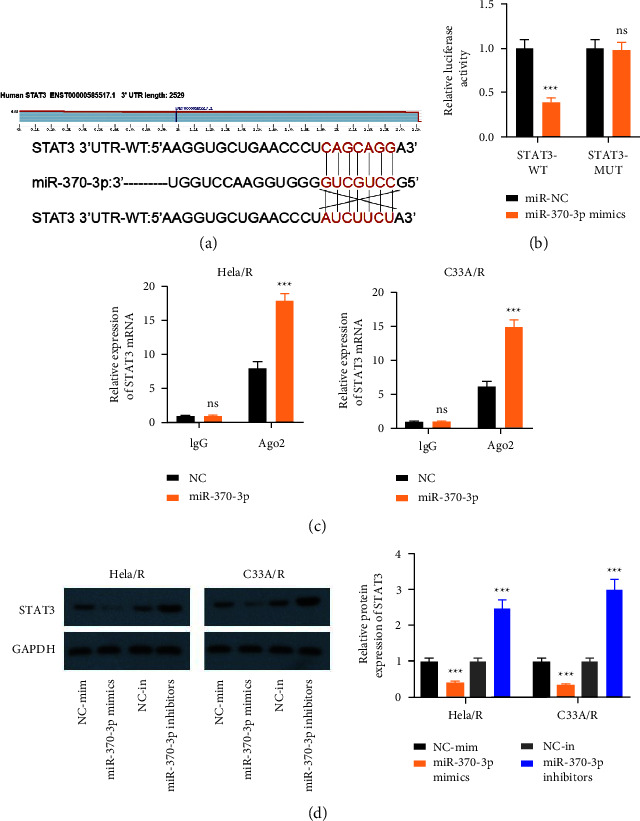
STAT3 was the downstream target of miR-370-3p. (a) There were conservative binding sites between STAT3 and miR-370-3p. (b) Dual-luciferase reporter assay was used to detect the binding of MALAT1 to miR-370-3p. (c) RIP assay to detect the combination of STAT3 and miR-370-3p. (d) The level of STAT3 after regulation of miR-370-3p was detected by western blot. ^*∗*^*P* < 0.05, ^*∗∗*^*P* < 0.01, ^*∗∗∗*^*P* < 0.001. All experiments were performed at least three times.

More and more evidence shows that miRNAs can perform their function by targeting combined with mRNA. Thus, mRNA that might be a objective of miR-370-3p was predicted by TargetScan. The data showed that miR-370-3p might interact directly with STAT3 (Figure 5(a)). This result showed that co-transfection with wild-type STAT3 (WT-STAT3) plasmid and miR-370-3p mimics markedly decreased the luciferase activity of HeLa/R and C33A/R cells, while co-transfection with mutant STAT3 (MUT-STAT3) plasmid and miR-370-3p mimics did not change the luciferase activity (Figure 5(b)). In addition, AGO2-containing beads were significantly enriched in miR-370-3p and STAT3 (Figure 5(c)). Furthermore, STAT3 decreased in HeLa/R and C33A/R cells, while transfected miR-370-3p mimics, demonstrating the target binding relationship between miR-370-3p and STAT3 (Figure 5(d)). Hence, these data indicated that STAT3 was the target of miR-370-3p.

### 3.6. MALAT1 Promoted Cisplatin Resistance of CC Cells by Up-Regulating STAT3 and Activating PI3K/AKT Pathway by Adsorbing miR-370-3p

**Figure 6:**
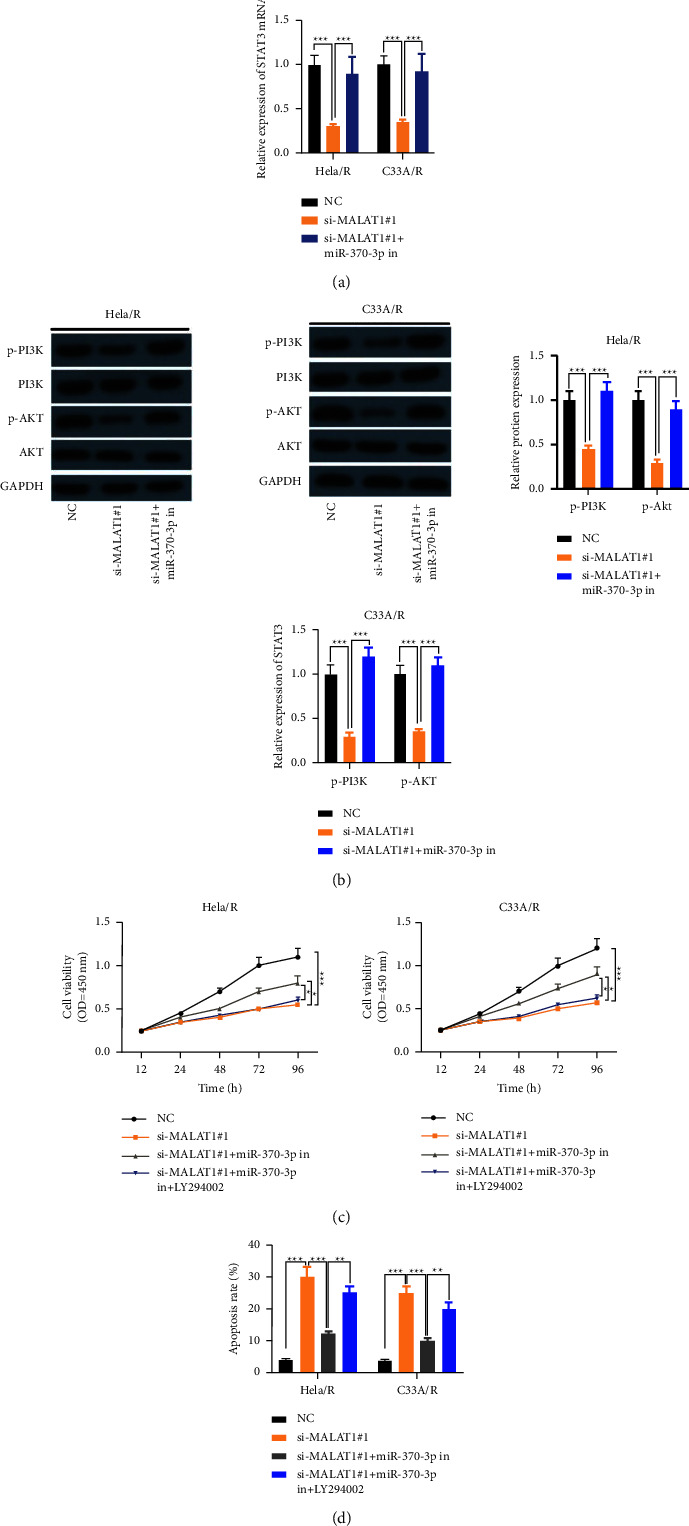
MALAT1 up-regulated STAT3 and activated PI3K/AKT pathway by adsorbing miR-370-3p to promote cisplatin resistance in CC cells. MALAT1 low expression plasmid and miR-370-3p inhibitors were co-transfected into Hela/R and C33A/R cells. (a) qRT-PCR was used to detect the level of STAT3. (b) The protein levels of phosphorylated PI3K (p-PI3K), total PI3K, phosphorylated AKT (p-AKT) and total AKT were detected by the Western blot method. Pretreatment of CC cisplatin-resistant cells with PI3K/AKT specific inhibitor LY294002. (c) CCK-8 assay was used to detect cell proliferation. (d) Apoptosis was detected by flow cytometry. ^*∗*^*P* < 0.05, ^*∗∗*^*P* < 0.01, ^*∗∗∗*^*P* < 0.001. All experiments were performed at least three times.

To explore the mechanism of MALAT1 regulating cisplatin resistance, we co-transfected MALAT1 knockdown plasmid and miR-370-3p inhibitors into Hela/R and C33A/R cells. MALAT1 silencing down-regulated the level of STAT3, while inhibition of miR-370-3p reversed its level (Figure 6(a)). As shown in Figure 6(b), the expression of p-PI3K and p-AKT in MALAT1-low expression CC cisplatin-resistant cells was substantially lower than that in the control group, while the transfection of miR-370-3p inhibitors p-PI3K and p-AKT in CC cisplatin-resistant cells was significantly up-regulated. These results suggest that MALAT1 activates the PI3K/AKT pathway in CC-resistant cells by regulating the miR-370-3p/STAT3 axis. CC cells resistant to cisplatin were pretreated with LY294002, a specific PI3K/AKT inhibitor. CCK-8 assay proved that compared with MALAT1+miR-370-3p in the roup, the proliferation ability of Hela/R and C33A/R cells was inhibited by LY294002 treatment (Figure 6(c)). To further confirm this result, we carried out an apoptosis experiment. In the presence of LY294002, the apoptosis of CC-resistant cells was higher than that of MALAT1+miR-370-3p in the group (Figure 6(d)). These results suggest that MALAT1 up-regulates the STAT3-activated PI3K/AKT pathway by adsorbing miR-370-3p to enhance cisplatin resistance in CC cells.

### 3.7. Exosomal MALAT1 Might be a Promising Biomarker for the Diagnosis of Cisplatin-Resistant CC Patients

**Figure 7:**
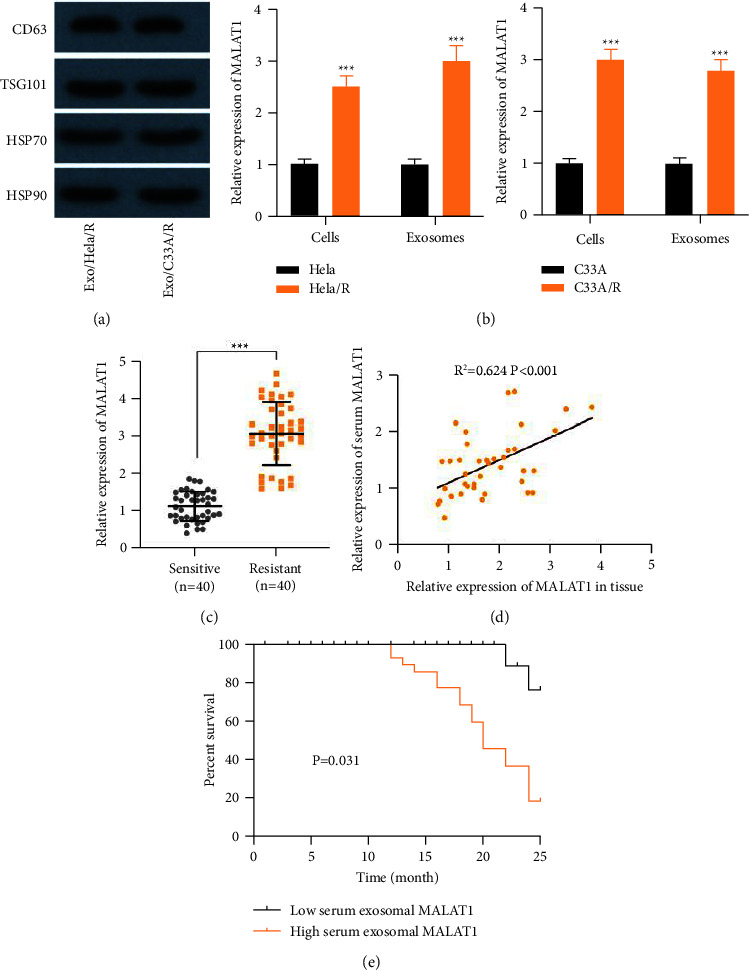
Exosome MALAT1 might be a promising biomarker for the diagnosis of cisplatin-resistant CC patients. (a) Western blot analysis of Exo/Hela/R and Exo/C33A/R exosome markers CD63, TSG101, HSP70, and Hsp90. (b) The expression of MALAT1 in CC cells and exocrine bodies were analyzed by real-time quantitative PCR. (c) The expression of MALAT1 was detected in the extracted serum exosomes, and the expression in the cisplatin-resistant group was higher than that in the cisplatin-sensitive group. (d) Pearson's analysis was used to analyze the correlation of MALAT1 expression in tissue and serum. (e) The relationship between the level of serum exosomal MALAT1 and the survival rate of patients. ^*∗∗∗*^*P* < 0.001. All experiments were performed at least three times.

**Figure 8:**
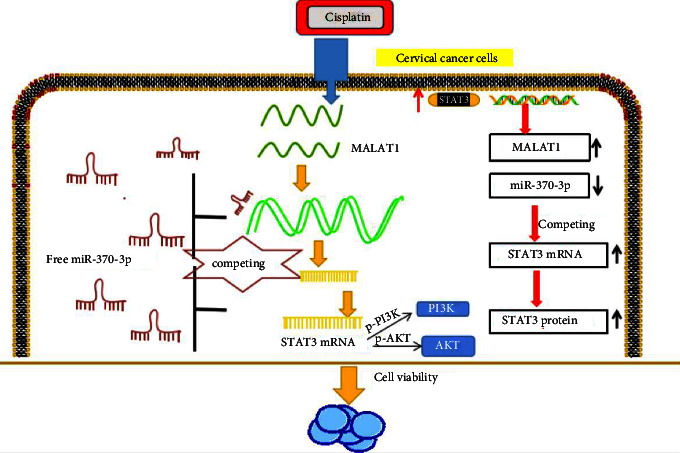
Exosomal lncRNA MALAT1/miR-370-3p/STAT3 loop affected PI3K/AKT pathway mediated the cisplatin resistance of CC cells.

Subsequently, this study explored the mechanism of cisplatin resistance based on exosomes. We isolated and identified exosomes from Hela/R and C33A/R cells. Western blot analysis confirmed the existence of four well-known exosome markers, CD63, TSG101, HSP70, and HSP90 (Figure 7(a)). This showed that the exosome was separated successfully. We observed the MALAT1 expression in Hela/R and C33A/R cells treated with or without exosomes increased markedly (Figure 7(b)). In addition, we extracted exosomes from serum samples of 80 CC patients treated with MALAT1. The results showed that MALAT1 could be detected in the extracted serum exosomes, and the expression of MALAT1 was higher in the MALAT1 resistant group than that in the MALAT1 sensitive group. (Figure 7(c)). Moreover, we detected the MALAT1 expression in exosomes extracted from tissue and serum showed a positive correlation (Figure 7(d)). CC patients with higher serum exosome MALAT1 levels have a poor prognosis, indicating adverse reactions to Cisplatin treatment (Figure 7(e)). Our data suggested that exosomal MALAT1 might seem like a promising diagnostic biomarker for CC patients. Finally, Figure 8 summarizes the speculative mechanism diagram including MALAT1, miR-370-3p, STAT3, and PI3K/Akt pathway.

## 4. Discussion

As a first-line high-efficiency broad-spectrum antineoplastic drug, cisplatin is the most basic drug for the treatment of cancer and is widely used, but cisplatin is a cytotoxic compound, which lacks selectivity to cancer cells and is easy to cause a lot of severe adverse reactions. The emergence of cisplatin resistance to chemotherapy has become a major restriction in treating cancer patients [22, 23]. More and more reports have revealed the molecular and cellular mechanisms of cisplatin resistance in many kinds of tumors [24, 25]. Furthermore, exosomal lncRNA is considered to play a vital role in chemotherapy resistance of many cancers [26]. We quested the part of exosomal MALAT1 in cisplatin resistance in CC cells and its mechanism. The abundance of MALAT1 increased in cisplatin-resistant CC cells. Besides, STAT3 may up-regulate the expression of cisplatin-resistant CC cells. It is further confirmed that the exosomal lncRNA MALAT1/miR-370-3p/STAT3 loop affected PI3K/AKT pathway mediated the cisplatin resistance of CC cells (Figure 8).

It is reported that the abnormal MALAT1 expression is closely associated with the chemoresistance of different cancers. For instance, MALAT1 was increased in gastric cancer and acts as an oncogene to enhance the resistance to oxaliplatin [27]. MALAT1 in bladder cancer is increased, and its overexpression contributed to cisplatin resistance [28]. Similarly, MALAT1 promoted cisplatin resistance in cervical cancer, which might be attributed to the inhibition of apoptosis by activating the PI3K/AKT pathway [12]. The abundance of MALAT1 in cisplatin-resistant CC cells increased, and it was related to poor clinical prognosis. Its knockout could inhibit the proliferation and promote the apoptosis of cisplatin-resistant CC cells. This suggests that MALAT1 is necessary to maintain cisplatin resistance in CC cells. Furthermore, exosomes have been recognized to be involved in the regulation of cellular communication and receptor cell chemosensitivity [29]. Previous studies have shown that the expression of exosome MALAT1 in CC is usually up-regulated [13]. Our study explored the mechanism of cisplatin resistance based on exosomes and isolated and identified exosomes from Hela/R and C33A/R cells. We found that exon protein markers were effectively expressed in exosomal, and the expression of MALAT1 from the extracted exon was enhanced in cisplatin-resistant CC cells. In addition, the expression could be detected in the extracted serum exosome, and the expression was higher in the MALAT1 resistant group. We also demonstrated that CC patients with higher serum exosomal MALAT1 levels had a poor prognosis, indicating adverse reactions to cisplatin therapy. Thus, these findings suggest that exosomal MALAT1 promotes cisplatin resistance in CC cells and that external MALAT1 served as a promising diagnostic biomarker in patients with cisplatin-resistant CC.

Recent studies have shown that the mutual regulation between lncRNA and miRNA plays an essential part in the occurrence of tumor drug resistance. MALAT1 regulates chemotherapy resistance related to autophagy in gastric cancer by targeting miR-23b-3p [30]. At the same time, MALAT1 regulates the miR-3129-5p/Nova1 axis to reduce the sensitivity of hepatocellular carcinoma to adriamycin [31]. In CC, lncRNA PVT1 regulated miR-195 to enhance the paclitaxel resistance in cervical cancer [32]. Besides, previous studies have confirmed that MALAT1 targeting miR-370-3p attenuates LPS-induced acute renal injury [17]. Therefore, we explored whether MALAT1 can be used as a competing endogenous RNAs (ceRNAs) to regulate miRNAs in CC cisplatin resistance. In the present study, through bioinformatics analysis, it was predicted that MALAT1 contained the binding sequence of miR-370-3p and confirmed the combination of the two. According to previous studies, miR-370-3p was involved in chemoresistance. For example, miR-370-3p inhibits chemotherapy resistance to glioblastoma by targeting MGMT [33]. Moreover, former studies have proved that miR-370-3p was decreased in cervical cancer [16]. Consistent with the above studies, miR-370-3p was down-regulated in CC cisplatin-resistant patients and drug-resistant cells. miR-370-3p could partially reverse the role of MALAT1 in promoting cell activity and inhibiting apoptosis in CC drug-resistant cells, indicating that MALAT1 regulates cisplatin resistance in CC cells by targeting miR-370-3p.

STAT3 disorders are associated with drug resistance phenotypes. For example, the STAT3 pathway regulates the proliferation and migration of drug-resistant bladder cancer cells by regulating Cyclin D1 and MMP2 [34]. In CC, lncRNA MALAT1 attenuates cisplatin-induced apoptosis of cervical cancer cells by regulating the STAT3 signal through miR-21 [35]. Studies have confirmed that STAT3 could regulate the level of lncRNA as a transcription factor. Such as, in hepatocellular carcinoma, STAT3-mediated lncRNA HOXD-AS1 acts as ceRNA to promote the metastasis of hepatocellular carcinoma [36]. To quest whether the expression of MALAT1 in cisplatin-resistant CC cells was mediated by STAT3, the online transcription factor program was utilized to predict the binding of the MALAT1 promoter region to STAT3. In addition, the expression of STAT3 in cisplatin-resistant CC cells was up-regulated. Further experiments confirmed the enrichment of STAT3 in the MALAT1 promoter region. Many reports have proved that lncRNAs may act as ceRNAs to regulate the target mRNA of miRNAs in tumors [37]. We would like to know whether MALAT1 acts as a ceRNAs to regulate cisplatin resistance in CC cells. Interestingly, bioinformatics analysis predicted hypothetical binding sites between miR-370-3p and MALAT1 or STAT3, and this prediction was confirmed by dual-luciferase reporter analysis and RIP analysis. Functional experiments further proved that MALAT1 up-regulated STAT3 by adsorbing miR-370-3p to promote cisplatin resistance of CC cells. PI3K/Akt signaling pathway is abnormally activated in most malignant tumors and plays a vital function in cell proliferation and drug resistance [38]. Studies have shown that the expression of STAT3 is up-regulated in ovarian cancer tissues, and the resistance of ovarian cancer cells to cisplatin is reduced through PI3K/AKT pathway [19]. We found that MALAT1 could up-regulate STAT3 and activate PI3K/AKT pathway by adsorbing miR-370-3p to enhance the cisplatin resistance of CC cells. Therefore, we want to know whether the MALAT1/miR-370-3p/STAT3 axis can regulate the PI3K/AKT pathway. MALAT1 activated the PI3K/AKT pathway in CC-resistant cells by regulating the miR-370-3p/STAT3 axis. Moreover, through the pretreatment of PI3K/AKT specific inhibitor (LY294002), the cell function assay further certified the capacity of MALAT1/miR-370-3p/STAT3 in CC cisplatin-resistant cells by activating PI3K/AKT pathway.

To sum up, these findings suggested that the exosomal MALAT1/miR-370-3p/STAT3 loop affects the PI3K/AKT pathway mediating the cisplatin resistance of cervical cancer cells, which provides a promising exosomal lncRNA targeting therapy for CC patients and an important theoretical basis for CC therapy. Our results provide new insights and help identify new prognostic biomarkers and treatment targets, which may help clinicians predict the prognosis of cisplatin-resistant patients with cervical cancer and inform their treatment decisions.

## Figures and Tables

**Table 1:** PCR primer sequences.

cDNA	Primer sequences (5′–3′)
MALAT1 (F)	TGCGAGTTGTTCTCCGTCTA
MALAT1 (R)	TATCTGCGGTTTCCTCAAGC
miR-370-3p (F)	GCCTGCTGGGGTGGAACCTGGT
miR-370-3p (R)	GCAGGGTCCGAGGTATTC
STAT3 (F)	ATCACGCCTTCTACAGACTGC
STAT3 (R)	CATCCTGGAGATTCTCTACCACT
*β*-actin (F)	CTGGGCTACACTGAGCACC
*β*-actin (R)	AAGTGGTCGTTGAGGGCAATG
U6 (F)	CGCAAGGATGACACGCAAAT
U6 (R)	GCAGGGTCCGAGGTATTC

**Table 2:** Correlation between MALAT1 expression and clinicopathological characteristics of CC patients.

Clinicopathologic features	Total	MALAT1 expression		*P* value
		High (*n* = 40)	Low (*n* = 40)	
Age (years)				0.073
<45	38	15	23	
≥45	42	25	17	

Tumor size				
<4 cm	38	12	26	0.001
≥4 cm	42	28	14	

FIGO stage				0.001
I-II	41	10	31	
III-IV	39	30	9	

Lymph node metastasis				0.004
Yes	28	20	8	
No	52	20	32	

Note. ^*∗*^*P* < 0.05 means the difference is statistically significant.

## Data Availability

The datasets used and/or analyzed during the current study are available from the corresponding author on reasonable request.
